# Correction: Culturing and patch clamping of Jurkat T cells and neurons on Al_2_O_3_ coated nanowire arrays of altered morphology

**DOI:** 10.1039/c9ra90030f

**Published:** 2019-05-02

**Authors:** Jann Harberts, Robert Zierold, Cornelius Fendler, Aune Koitmäe, Parisa Bayat, Irene Fernandez-Cuesta, Gabriele Loers, Björn-Philipp Diercks, Ralf Fliegert, Andreas H. Guse, Carsten Ronning, Gaute Otnes, Magnus Borgström, Robert H. Blick

**Affiliations:** Center for Hybrid Nanostructures, Universität Hamburg Luruper Chaussee 149 22761 Hamburg Germany rblick@chyn.uni-hamburg.de +49 40 42838 1975; Center for Molecular Neurobiology Hamburg, University Medical Center Hamburg-Eppendorf Falkenried 94 20251 Hamburg Germany; Department of Biochemistry and Molecular Cell Biology, The Calcium Signaling Group, University Medical Center Hamburg-Eppendorf Martinistraße 52 20251 Hamburg Germany; Institute for Solid State Physics, Friedrich-Schiller-University Jena Helmholtzweg 3-5 07743 Jena Germany; NanoLund, Lund University Box 118 22100 Lund Sweden; Solid State Physics, Lund University Box 118 22100 Lund Sweden; Material Science and Engineering, College of Engineering, University of Wisconsin-Madison Madison Wisconsin 53706 USA

## Abstract

Correction for ‘Culturing and patch clamping of Jurkat T cells and neurons on Al_2_O_3_ coated nanowire arrays of altered morphology’ by Jann Harberts *et al.*, *RSC Adv.*, 2019, **9**, 11194–11201.

The authors regret that some of the figure citations in the text were incorrect in the original article. The correct figure citations are as follows. On page 11197 the sentence beginning, “For neurons…” should read, “For neurons, shown in Fig. 2d, we observed cell viabilities of 73.9 ± 3.6% and 72.9 ± 6.2% on the control substrates and 65.4 ± 4.5% and 66.5 ± 4.4% on the NW substrates.” Additionally, the sentence beginning, “Furthermore, exemplary images…” should read, “Furthermore, exemplary images of stained neurons cultured on both types of NWs are shown in Fig. 2e and f as well as on the control substrates in Fig. S2.”

The Royal Society of Chemistry apologises for these errors and any consequent inconvenience to authors and readers.

## Supplementary Material

